# Updated guidelines for the reporting of methods and statistical analyses

**DOI:** 10.1111/aogs.14760

**Published:** 2023-12-28

**Authors:** Philip M. Sedgwick

**Affiliations:** ^1^ Institute for Medical and Biomedical Education, St. George's, University of London London UK

*Acta Obstetricia et Gynecologica Scandinavica (AOGS)* has refined and expanded its guidelines for authors when reporting methods and statistical analyses. Their development reflects recent advancements and thinking in research methodology. The guidelines also address the increasing concern in the research community regarding the misuse and misinterpretation of statistical hypothesis testing, more formally known as null hypothesis significance testing (NHST).

*AOGS* is a member of the Committee on Publication Ethics (COPE). As part of its strategic plan, the purpose of COPE is to “Educate and advance knowledge in methods of safeguarding the integrity of the scholarly record.”^1^ The development of robust guidelines will help support the core principles of COPE, namely transparency and best practice in scholarly publishing. By encouraging authors to report their research in a consistent and standardized manner, reviewers will be able to evaluate more easily the worth and credibility of the submitted manuscript. Furthermore, by bringing clarity and accuracy to reporting it will enhance the readers' understanding of the research conducted and results obtained.

Development of the guidelines was in accordance with the recommendations of the International Committee of Medical Journal Editors (ICMJE). ICMJE proposed the following aims and objectives for a methods section incorporating the statistical methods. The methods section “… should aim to be sufficiently detailed such that others with access to the data would be able to reproduce the results”.^2^ Furthermore, the statistical methods should be described “…with enough detail to enable a knowledgeable reader with access to the original data to judge its appropriateness for the study and to verify the reported results”.^2^

The updated guidelines cover many aspects of the reporting of experimental (interventional) and observational studies. These are embedded within those of the EQUATOR (Enhancing the QUAlity and Transparency Of health Research) network.^3^ The network is an international initiative which “seeks to improve the reliability and value of published health research literature”. The updated journal guidelines should be seen to supplement the guidance of the network, and not replace it. The EQUATOR network has brought together published statements providing guidelines for the design, planning, and reporting of many different study designs, plus extensions for specialized designs and specific topics. The statements were developed to enhance the transparency and standardization of the presentation of methods and statistical analyses, thereby promoting the comparison with similar research. Authors must ensure they adhere to these statements when reporting their research. Many of the statements incorporate a checklist, indicating the list of items that should be reported in a manuscript. Authors should include a completed checklist, indicating where in their manuscript they have reported each of the required items. Some statements also promote the completion of a flow diagram indicating the progression of participants through the study. Where appropriate, authors are encouraged to include a completed flow diagram in their submission.

There is a focus in the updated guidelines on the reporting and interpretation of results using traditional statistical hypothesis testing. Journal editors, statisticians, and the wider research community are becoming increasingly concerned about the misuse and misinterpretation of NHST as based on the dichotomy of statistical significance (*p* < 0.05 vs. *p* ≥ 0.05). Such an approach has little foundation and encourages a cookbook recipe approach to decision‐making. It was never intended for clinical significance to be inferred from statistical significance (*p* < 0.05), or for the lack of clinical importance otherwise (*p* ≥ 0.05). The debate about the misuse of NHST has intensified within the last decade. In 2016, the American Statistical Association published a statement stressing the need for the proper use and interpretation of *p*‐values.^4^ Shortly afterwards, a prominent article was published in *Nature* calling for the abandonment of decision‐making based on statistical significance.^5^ It was claimed it had led to “…hyped claims and the dismissal of possibly crucial effects”. Members of the research community have responded to this call for a statistics reform. Some medical journals now discourage the use of statistical significance in decision‐making. Moreover, authors are encouraged to place greater emphasis on the interpretation of the magnitude of absolute effect sizes, plus their associated 95% confidence intervals.

*AOGS* is not banning statistical hypothesis testing, *p*‐values, and inferences based on statistical significance (*p* < 0.05). However, it is imperative that *p*‐values are never interpretated without inspection of the absolute effect estimates plus their 95% confidence intervals. In the presence of statistically significant results (or otherwise), authors should consider the potential implications for future research, clinical practice, or health policy. Authors must also acknowledge the further limitations of traditional NHST as discussed in a recent commentary in *AOGS*.^6^ The major shortcomings of NHST are type I and II errors which lead to, for example, false claims about the effectiveness of interventions or dismissal of potentially important ones. The challenges of multiplicity, whereby multiple hypothesis testing increases the probability of type I errors occurring, must be addressed. Authors should also consider the implications of sample size. As sample size increases, increasingly smaller differences between groups will become statistically significant. Hence, NHST ensures that any difference between groups, regardless of how small or irrelevant it is, will be statistically significant if sample size is large enough.^7^

Authors are encouraged to report whether their research is exploratory or confirmatory by design. Nonetheless, it is appreciated that such designs exist on a continuum rather than as a dichotomy. Exploratory research, often subdivided into feasibility and pilot studies, explores the feasibility and uncertainties of undertaking a future larger confirmatory randomized controlled trial or observational study.^8^ Exploratory research is typically concerned with generating hypotheses, which are subsequently tested in confirmatory research using NHST. Sample sizes in exploratory research tend to be small and consequently lack statistical power to test, for example, the effectiveness of interventions or importance of exposure to risk factors. For that reason, statistical hypothesis testing is not usually undertaken in exploratory research. If statistical hypothesis testing is undertaken, authors must interpret their results with caution.^9^ Authors reporting exploratory research should make great efforts to stress that all findings are reported as tentative and hypothesis generating, rather than hypothesis testing.

The distinction between exploratory and confirmatory research is not new. However, the application of exploratory research with restricted use of NHST has received increased attention recently. It is suggested the current drive for the informed use of NHST has contributed to this. Authors should not consider exploratory research as being any less worthy than confirmatory research, simply because statistical hypothesis testing is not advocated. It is anticipated that authors will be less familiar with exploratory research methods. Authors are encouraged to read guidance on the definition of exploratory studies,^9^, ^10^ along with advice on suggested sample sizes in exploratory trials.^11^ Statements for reporting nonrandomized pilot and feasibility studies,^12^ plus randomized pilot and feasibility (exploratory) trials^13^ are referenced in the guidelines.

Authors must plan their data analyses in advance and have stated the planned analyses in their statistical analysis plan. Subgroup analyses, whereby the sample is broken into subsets of participants based on shared characteristics, will have reduced power whilst they are prone to the challenges of multiplicity. Therefore, any planned subgroup analyses requires a research strategy to enhance statistical power and control the type I error rate. Analyses that emerge during the planned analyses, including subgroup analyses, are considered exploratory with the aim of generating hypotheses. Therefore, for unplanned analyses statistical hypothesis testing is not normally undertaken.

Authors should refrain from questionable research practices such as *P*‐hacking and fishing expeditions.^14^ These involve persistently analyzing the data in different ways, for example based on subgroups or indiscriminately examining associations between different configurations of variables. Such practices will ultimately result in statistically significant findings. However, statistical significance will have arisen due to multiplicity, and not necessarily because of any important contextual differences.

The new guidelines focus on the reporting of statistical analyses and results stemming from the traditional statistical frequentist approach, not least because most submitted manuscripts use such methodology. However, manuscripts based on Bayesian methods are welcome. The journal is also keen for the submission of manuscripts reporting qualitative research. If authors use unusual or innovative methods, they should describe them briefly so that readers can appreciate the aims and objectives of the techniques. Similarly for approaches not frequently used, for example Bayesian methods, the need for brief descriptions clarifying terminology should be considered. If authors wish, they can provide detailed descriptions of methods as supplementary material for the interested reader.

Authors are strongly encouraged to read the guidelines and adhere to them when preparing a manuscript for submission. If authors have already submitted a manuscript they may be asked to adhere to the new guidelines. By doing so, authors will experience a more efficient and quicker review process. However, it is recognized the updated guidelines may present challenges for authors. Some may be accustomed to certain practices, for example the direct inference of clinical significance from statistical significance. Furthermore, some studies may have been designed and undertaken some time ago, making it difficult for them to be reported in accordance with a statement for a particular study design. Therefore, a period of transition may be necessary whereby editors and authors work together. Nonetheless, it is hoped that authors will appreciate the guidelines have been developed to encourage scientific integrity, thereby promoting unequivocal confidence and trust in the research published in *AOGS*.
